# Salivary Metabolites Produced by Oral Microbes in Oral Diseases and Oral Squamous Cell Carcinoma: A Review

**DOI:** 10.3390/metabo14050277

**Published:** 2024-05-10

**Authors:** Bina Kashyap, Arja Kullaa

**Affiliations:** Institute of Dentistry, University of Eastern Finland, 70211 Kuopio, Finland; bina.kashyap@uef.fi

**Keywords:** dysbiosis, oral microbiome, saliva, metabolites, oral disease, oral cancer

## Abstract

In recent years, salivary metabolome studies have provided new biological information and salivary biomarkers to diagnose different diseases at early stages. The saliva in the oral cavity is influenced by many factors that are reflected in the salivary metabolite profile. Oral microbes can alter the salivary metabolite profile and may express oral inflammation or oral diseases. The released microbial metabolites in the saliva represent the altered biochemical pathways in the oral cavity. This review highlights the oral microbial profile and microbial metabolites released in saliva and its use as a diagnostic biofluid for different oral diseases. The importance of salivary metabolites produced by oral microbes as risk factors for oral diseases and their possible relationship in oral carcinogenesis is discussed.

## 1. Introduction

Globally, the burden of oral cancers is rapidly increasing in various regions of the world. Worldwide, 354,864 new cases of oral cancer and 177,384 estimated cancer deaths per year were reported [1]. The prevalence of oral cancer has been detected to be highly variable according to age, gender, diet, etiological factors, and geography [1]. Oral squamous cell carcinoma (OSCC) is the most frequently occurring cancer in the oral cavity, arising from the mucosal surfaces. These cancers represent a heterogeneous disease group with high rates of recurrence, and frequently undergo lymph node metastasis [2]. Though the recent advancement in treatment of OSCC has improved, the early diagnosis is prioritized for better prognosis. An emerging concept in cancer implicates that oral microbiome is an influential factor that modulates the carcinogenic process. Hence, for the past few years, research has been undertaken to explore the oral flora, which has multiple protective physiological functions along with nutritional and detoxification functions [3].

According to Human Oral Microbiome Database (HOMD), the oral cavity harbors more than 770 species, with more than half having formal names (58%), a few being unnamed but cultivated (16%), and the rest being uncultivated phylotypes (26%) [4]. The oral microbiome plays an important role in the metabolism of the oral cavity, and the end-product produced by bacterial metabolism is either absorbed by oral epithelial cells or remains in the oral fluids. It is difficult to relate bacterial diversity in patients with different oral diseases, even though many diseases are caused due to alterations in microbial composition. The oral microbiome that alters the saliva metabolism profile has been investigated in oral and systemic diseases [5]. Human oral microbiome studies use the 16S rRNA-based next generation sequencing (NGS) method to the functional and structural aspects of bacterial communities in healthy and diseased conditions. Alterations in the oral microbiome causes inflammation that accelerates OSCC through direct metabolism of carcinogenic substances [6,7].

Saliva is a complex biological fluid that contains a broad spectrum of biomarkers of health and disease status [8,9,10]. Salivary metabolites, produced by oral microorganisms, present the changes in the oral metabolic pathways and, hence, it is suggested as a potential source of biomarkers to assess oral diseases [11]. Nuclear magnetic resonance (NMR) spectroscopy, mass spectrometry (MS), gas chromatography (GS), capillary electrophoresis (CE) or high-performance liquid chromatography (HPLC) are methods used to analyze salivary metabolites [12]. Studies on salivary metabolomics have provided information on biochemical pathways involved in oral diseases such as dental caries, periodontitis, oral premalignancy and oral squamous cell carcinoma [11]. Hence, oral microbes contribute to the salivary metabolic fingerprint and the oral microbiome is one of important sources of salivary metabolites [11]. This narrative review focuses on changes in oral conditions where oral commensal bacteria become pathological and produce harmful metabolites. In addition, healthy oral bacterial microbiome and their impact on the oral biofilm and host are presented. We also describe some significant biochemical metabolic pathways that contribute to OSCC progression.

## 2. Healthy Commensal Oral Microbiome

**Figure 1:**
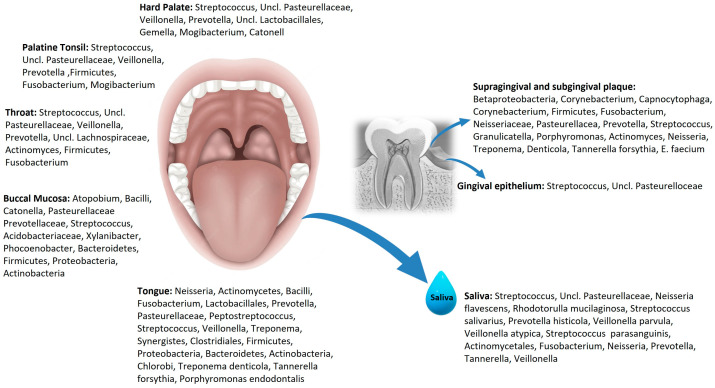
Site-specific oral microbial distribution in the oral cavity.

The oral cavity presents different niches for millions of micro-organisms to colonize, including bacteria, fungi, viruses, protozoa, and archaea. The oral microbiome, together with saliva, plays a key role in the oral homeostasis between health and diseases. It is suggested that the organisms inhabiting saliva construct an optimal environment for their survival by the absorption of nutrients and interaction through quorum sensing to regulate their growth rates [13]. The HOMD lists site-specific oral bacterial composition that are not uniformly distributed over all surfaces but can proliferate differently in ecological niches depending on their metabolism in a healthy oral cavity (Figure 1) [14,15].

The tongue, buccal mucosa, throat, tonsils, palate, tooth surfaces, gingival pocket and saliva of the oral cavity represent different ecological niches or habitats. The tongue presents highest diversity of microbiome, and facilitates bacterial colonization in other regions of the oral cavity through saliva [16]. Exfoliated oral epithelial cells with attached bacteria are observed in saliva [17]. Some bacterial species pose different receptors and adhesion molecules to ensure colonization on different surfaces in the oral cavity such as the teeth, tongue, or mucosa. Streptococcus, a heterogeneous group, is the most common genera found in oral cavity, which can colonize on hard tissue, soft tissue, and is present in saliva [18]. HOMD lists *firmicutes* (genera *streptococcus* and *granulicatella*), *Actinobacteria* (genera *corynebacterium*, *Rothia*, *Actinomyces*), *Fusobacteria* (genera *fusobacterium*), *Bacteroidetes* (genera *Prevotella*, *Capnocytophaga*, *Porphyromonas*) and *Proteobacteria* (genera *Neisseria*, *Haemophilus*) as most prevalent phyla of adult human oral cavity [19]. A reliable relationship was established between oral microbiome and systemic diseases, including cardiovascular diseases [20], neurodegenerative diseases [21], rheumatoid arthritis [22], preterm birth [23], cancers [7] and inflammatory bowel disease [24].

Apart from bacteria, some diverse non-bacterial forms of oral microbes are protozoa (mainly *Entamoeba gingivalis* and *Trichomonas tenax*), fungi (species Candida mainly Candida albicans) and viruses, including herpes simplex virus (HSV), human papilloma virus (HPV), Epstein–Barr virus (EPV), and human immunodeficiency virus (HIV) [7]. The presence of several phages in the salivary and dental plaque samples was identified, belonging to the family of *Siphoviridae*, *Myoviridae* and *Podoviridae*, respectively [25]. These phages are reported to be quite stable in the oral cavity and the inactivation of the bacterial defense mechanisms leads to their establishment in the oral cavity. A study on oral bacteriophages confirmed it to be highly individual and gender specific compared with other habitats inside the human body like gut and skin, etc. [26]. Oral cavity phages are also associated with diseases like endocarditis due to presence of high number of virulence genes [27]. Candida species is the most prevalent form in the oral cavity and is primarily responsible for different oral infections [28]. Viruses are also associated with periodontitis along with bacteria [29]. HPV is associated with some oral disorders, including condylomas, papillomas, epithelial hyperplasia and head and neck squamous cell carcinoma [30].

## 3. Oral Biofilm and Oral Microbial Metabolites

The oral health of an individual depends on the presence of healthy biofilms on the surfaces of the gums, teeth, and mucosal linings of the oral cavity. Changes in microbial density in the oral cavity depend on the interaction between different microbial species in the biofilm. Oral biofilms are complex with different microbial species, proteins, lipids, carbohydrates, salivary and host components. The formation, development and maturation of oral biofilm occurs through the complex symbiotic interactions of different microbes. This includes mechanisms, involving coagulation, metabolic exchange, communication, and exchange of genetic material [31]. Quorum sensing (QS) has been widely used by many bacterial and fungal species especially to regulate biofilm development and maintenance [32]. Different bacteril species of the oral cavity such as *Streoptocoocus* (*Streptococcus mutans*, *Streptococcus gordanii* and *Streptococcus mitis*) produce bacteriocins through quorum sensing and regulate the biofilm formation. Bacteriocin produced accounts for biodiversity and ecological suitability of microbes [33]. For example, *Streptococcus gordanii* can produce hydrogen peroxide, which prevents the growth of invading bacteria, thereby minimizing dental plaque formation. Also, hydrogen peroxide produced by *Streptococcus gordonii* is fatal for the growth of *Actinomyces naeslundii*, which is an important species in oral biofilms that remove hydrogen peroxide and aid in the growth of *Streptococcus gordonii* [34]. Other interactions reported in oral epithelial cells are between *Fusobacterium nucleatum* with *Streptococcus cristatus* [35].

Dental biofilms were studied and reported for different associations like coaggregation and metabolic cooperation with the food chains of various species [36]. Exposure to the fermentable carbohydrates alters the environment of microbiome in the oral cavity and affects the configuration and constituents of dental biofilms. The fermentation and production of metabolites favor the accumulation of pathogenic bacteria in the oral biofilms that contribute to development of the dental caries, periodontitis, and oral cancer [37]. *Streptococcus mutans*, *Lactobacilli*, *Bifidobacterium* subspecies, *Scardovia* subspecies, and *Actinomyces* subspecies are cariogenic bacteria. Saliva maintains the acidic environment of cariogenic biofilms in the oral cavity, which further enhances the demineralization of enamel by cariogenic bacteria [38]. *Streptococcus mutans* of cariogenic bacteria are responsible for the synthesis of glucans, an extracellular polymeric substance that allows more bacteria to bind through their surface proteins. The involved bacterial products and enzymes affect the sucrose-dependent biochemical pathway that induces plaque formation [39].

The primary nutritive source for the bacteria in supragingival and subgingival oral biofilm is saliva. Actinomyces subspecies and oral Streptococci (*Staphylococcus intermedius* and *Streptococcus oralis*) are the early colonizers in oral biofilms whereas *Porphyromonas gingivalis*, *Aggregatibacter actinomycetemcomitans*, *Prevotella intermedia*, *Eubacterium subspecies*, *Tannerella forsythia*, *Selenomonas flueggei*, and *Treponema denticola* are the late colonizers of the oral biofilm. *Fusobacterium nucleatum* aids in connecting early and late colonizers in the oral biofilms. Proline-rich salivary proteins and metabolic products, such as ammonia and organic acid, produced by bacteria help in interbinding of bacteria and pH maintenance in the biofilm [37,40]. So, most of the bacterial communities in the oral cavity of healthy individuals have pathogenic properties but, due to host tolerance, do not show symptoms.

The bacterial communities produce metabolites in the oral cavity that show alterations in the oral environment and biochemical pathways, and could be a potential factor in the pathogenesis of oral diseases. The anaerobic and saccharolytic bacteria in subgingival biofilm produce numerous metabolites, such as fatty acids (branched or short chain), amines and gases. This inflow of metabolites increases with increased activity and growth of the bacteria [41]. Many microbial metabolites, including ammonia, spermine, spermidine, hydrogen sulfide, and nitric oxide, have shown an increase in antibiotic resistance [42,43,44]. The oral microbiome and the released metabolites function in the periodontal tissue and their impact on the oral biofilm and the host are summarized in Table 1.

## 4. Oral Microbial Salivary Metabolites and Oral Diseases

Recently, the oral microbial contributions to the salivary metabolites have been appreciated in various oral diseases. The diagnostic utility of these salivary metabolites as biomarkers can reflect the changes in the oral microbiome. An imbalance in the oral microbiome (i.e., dysbiosis) is often associated with certain factors, such as the age of host, environmental factors (pH, temperature, nutrition in the oral cavity), host lifestyle (food habit, tobacco smoking, alcohol, oral hygiene, antimicrobial use) and changes in the salivary composition [70]. Oral diseases, like caries, gingivitis, periodontitis, and oral ulcerations, are related to oral microbial dysbiosis that produces metabolites to cause inflammation-mediated tissue destruction.

### 4.1. Dental Caries

A diet with an excess of carbohydrates produces acidic metabolites due to fermentation by the oral microbiome. This favor acidogenic and aciduric microorganisms that disturb the buffering capacity of saliva and, hence, cause dental caries. The genera *Veillonella*, *Bifidobacterium*, *Selenomonas*, *Olsenella*, *Parascardovia*, *Scardovia*, *Chryseobacterium*, *Terrimonas*, *Burkholderia*, *Neisseria*, and *Sporobacter* were highly observed in dental caries. *Veillonella*, with cariogenic potential, allows *Streptococcus* species to grow and produce acid that demineralizes tooth enamel [71]. Other species like *Prevotella*, *Lactobacillus*, *Dialister*, and *Filifactor* are involved in the pathogenesis and progression of dental caries [72]. Oral microbial metabolites such as lactate, acetate and n-butyrate have been observed in patients with dental caries. The reduction in salivary pH and increase porosity of the dental plaque matrix were related to the released metabolites in dental caries [73]. Associated with caries, salivary amino acid levels (proline and glycine) increase due to the hydrolysis of dentin-collagen [74]. Similarly, increased lipids on salivary pellicle can accelerate caries development by inhibiting acid diffusion [75]. Alanine, aspartate, glutamine, glycine, isoleucine, leucine, proline, taurine, tyrosine, fucose, galactose, glucose, xylose, choline, dimethylsulfone, hypoxanthine, menthol, N-acetyls, and uracil are the salivary metabolites observed in dental caries because of bacterial fermentative processes [76]. In a recent combined analysis of microorganisms and metabolites study, a significant correlation of the most differential salivary microorganisms with metabolites is observed in dental caries. *Veillonella*, *Staphylococcus*, *Streptococcus*, *Neisseria*, and *Porphyromonas* showed the most extensive correlations with metabolic differentials (mainly, 2-benzylmalate, epinephrine, 2-formaminobenzoylacetate, and 3-indoleacrylic acid). Among all, 2-benzylmalate, an organic acid metabolite, contributes to caries production via surface demineralization of dental tissues [77]. Hence, it can be speculated that the oral microbiome can significantly contribute to the salivary metabolome and can affect various biochemical pathways such as carbohydrate metabolism, organic acid metabolism, amino acid metabolism and other metabolic pathways and metabolites.

### 4.2. Periodontal Diseases

Oral microbiomes associated with the pathogenesis of periodontal diseases are *Prevotella intermedia*, *Fusobacterium nucleatum*, *Selenomonas noxia*, *Actinobacillus actinomycetemcomitans*, and *Eubacterium nodatum*, *Porphyromonas gingivalis*, *Treponema denticola*, and *Treponema forsythia*. These periodontopathogenic bacteria produce virulent factors such as lipopolysaccharides and peptidoglycans that can induce inflammation and tissue destruction [78]. The colonization of anaerobic bacteria in the periodontal pocket is assumed to have accumulated more diverse bacterial waste products due to the lack of salivary cleaning. Periodontal bacteria (mainly *P. gingivalis*, *Prevotella intermedia*, and *Fusobacterium nucleatum*) enable and maintain constant chronic inflammation and it contributes to OSCC development. Some periodontal pathogens have potential to affect the intracellular pathways and activate the oncogenic pathways [7,79]. Salivary metabolites produced by bacteria function as signaling molecules that can either trigger or inhibit the inflammatory response of the host. For example, the end-products of bacterial metabolism such as butyrate, caproate, isocaproate, propionate, isovalerate and lactate have been observed in saliva during inflammation and in deep periodontal pockets. The levels of these salivary metabolites are decreased after periodontal treatment and gradually increase over time. Hence, it is considered as possible indicators of periodontal disease development and progression [80].

The growing evidence on oral cancer and oral microbes has cleared that inflammation plays an important role in carcinogenesis. Interestingly, oral pathogenic bacteria have been shown to activate inflammatory pathways associated with cellular transformation. Some malignancies arise from the site of infection or inflammation, as a normal host response. Also, the literature shows that, globally, 1.2 million cases per year or 15% of malignancies worldwide are attributed to infections [79,81]. Periodontopathogenic bacteria, namely *P. gingivalis*, *Tannerella forsythia* and *Prevotella intermedia*, were involved in an increased risk of developing gastro-intestinal cancer [82]. Interestingly, these oral bacteria are not limited to oral cancers, but are also observed in the esophagus, stomach, pancreas, and colon/rectum tumors [83]. The periodontal bacteria, *P. gingivalis*, *Fusobacterium nucleatum*, *Alloprevotella* species, *Prevotella* species, *Capnocytophaga* species, *Streptococcus* species have been shown to be associated with progression and development of OSCC [84]. Patients with periodontal disease have shown an increase concentration of short chain fatty acids, breakdown the product of carbohydrates, proteins, and amino acids along with the predominance of *P. gingivalis* and *Treponema denticola* [85]. The metabolic product produced by the periodontal pathogen identified in various saliva metabolomic studies and are outlined in our previous article [11].

### 4.3. Oral Premalignancy

Oral leukoplakia (OL) and oral lichen planus (OLP) are oral potentially malignant disorders (OPMDs) [86], that are studied for oral microbiome and salivary metabolites. An abundance of *Fusobacterium nucleatum*, *Leptotrichia* species, *Campylobacter* species and *Rotha mucilaginosa* are observed in OL [87]. Apart from this, well-established periodontal pathogens mainly *Fusobacterium nucleatum*, *Prevotella intermedia* and *P. gingivalis*, are found to increase in OL [88]. Microbial metabolites like c-aminobutyric acid (GABA), phenylalanine, valine, lactate, eicosane, 4-nitroquinoline-1-oxide, are elevated in OL [11]. The increase in *Rhodotorula mucilaginosa* in OL suggests its role in the malignant transformation to OSCC via the production acetaldehyde, a toxic metabolite [89]. Individuals detected with acetaldehyde in saliva had shown increased levels of *Rhodotorula mucilaginosa* and *Streptococcus salivarius* in the salivary microbiome study [90].

Oral bacterial dysbiosis observed has shown high levels of *Porphyromonas*, *Solobacterium*, *Prevotella melaninogenica*, *Fusobacterium*, *Leptotrichia*, and *Lautotropia* in OLP [91,92]. *F. nucleatum*, a proinflammatory bacterium, is involved in the progression of OLP inflammation [93]. Indole-3-acetate and ethanolamine phosphate were elevated salivary microbial metabolites [94]. The oral microbial metabolites produced in premalignancy by *Fusobacterium*, *Prevotella*, *Porphyromonas*, *Veillonella*, *Actinomyces*, *Clostridium*, *Haemophilus*, *Streptococcus* subspecies, and *Enterobacteriaceae* are shared with OSCC [95]. Metabolic pathways, such as carbohydrate metabolism, amino acid metabolism, and organic acid metabolism are disturbed in patients with increased potential for malignant transformation, as OL and OLP [11].

### 4.4. OSCC

Oral microbes release salivary metabolites after multifactorial interactions between the host, oral bacteria, and altered cellular metabolism. The practical concerns to identify salivary metabolites mainly include standardized collection protocol and quality control of its components. Failure in standardization could lead to misleading connections between discovered markers and disease progression [96]. Most studies on oral microbiomes have proposed salivary metabolites as diagnostic indicators of oral cancer, but the search for a possible biomarker for OSCC has not given any convincing results. Different organisms have been shown to increase in the saliva samples of OSCC when compared with healthy controls presented in Table 2. The 16S rRNA or 16S rDNA genes are the part of DNA most used for bacteria. The widespread use of this gene sequence for bacterial identification and as a molecular chronometer is described by Woese [97]. This sequencing technique is more robust, reproducible, and accurate than phenotype testing or other techniques. Most of the studies on oral cancer have used 16S rRNA bacterial sequencing techniques (Table 2).

The presence of *Capnocytophaga*, *Prevotella*, *Sreptococcus*, and *Fusobacterium* species was significantly enriched in the saliva samples of OSCC patients (Table 2). The diagnostic sensitivity and specificity of approximately 80% for *Capnocytophaga*, *Prevotella*, and *Streptococcus* species in the saliva of OSCC patients was reported in a non-randomized study [98]. *Neisseria* species, in saliva can play a role in alcohol-related carcinogenesis by producing acetaldehyde [117]. Similarly, *P. gingivalis* was associated with advanced pathologic staging of OSCC, and *Fusobacteria* species were associated with significantly increased programmed death-ligand 1 (PD-L1) expression [118,119]. The mobile periodontal pathogens, *Fusobacterium nucleatum*, *Campylobacter* subspecies, *Pseudomonas aeruginosa*, and *Porphyromonas*, are observed in OSCC and are also associated with extra-oral infections and inflammation [84]. In a cell-based system and a mouse carcinogenesis model study, *Fusobacteria* have been shown to enhance the invasiveness, survival, and epithelial–mesenchymal transfer of cancer in the oral tumor microenvironment [120]. Likewise, *Porphyromonas gingivalis* and *Fusobacterium nucleatum* can initiate interleukin 9 (IL-9), tumor necrosis factor (TNF)-alpha, matrix metalloproteinases 1 and 9 (MMP-1 and 9) production and inhibition of apoptosis. Both *P. gingivalis* and *Fusobacterium nucleatum* have been shown to elevate the transcriptional activity of oncogenes and proinflammatory cytokines [121].

It has been reported that acidogenic and aciduric species can facilitate the invasion and metastasis of malignant cells. This occurs by promoting an acidic tumor microenvironment [122]. Oral microbial metabolites produced in the saliva of OSCC, implies the changed oral environment and an important factor in predicting OSCC prognosis. The various metabolites studied on OSCC (Table 3) can facilitate the diagnosis of conditions reflecting ecological dysbiosis, and suggest a change in the cellular biochemical metabolic pathway. The increased lactic acid and lower amino acid levels were correlated with increased glycolysis and impaired Krebs cycle in OSCC during cell proliferation [123].

Pathogenic bacteria can use pyrimidine metabolism to potentially alter the metabolic activity of the hosts and create oxidative stress and inflammation. Pyrimidine metabolism is involved in the synthesis, degradation, and interconversion of DNA, RNA, lipids, and carbohydrates. Purine degradation and altered pyrimidine metabolism are shown in salivary metabolomic studies on OSCC [94,127]. In OSCC, the low salivary concentration of urea implicates some dysfunction in the urea cycle [127,137]. Salivary urea regulates acid base balance in the oral cavity [144]. Hence, the changes in the concentration of urea in OSCC compromise acid base balance in oral environment.

N-Acetylglucosamine, one of the derivatives of glucose, is regulated in OSCC [94,124,126,131]. It can be linked to serine or threonine residues on the cytosolic and nuclear proteins in the form of O-linked β-N-acetyl glucosamine (O-GlcNAc). It is reported that the increased post-translational modification of O-GlcNAc in cancers is associated with transformed phenotypes [145]. Lactic acid, a product of glycolysis, is increased in OSCC, and it is associated with a decreased pyruvate entering tricarboxylic acid (TCA) cycle. Aerobic glycolysis in cancer cells is the main energy source [146]. The abundance of glucose in cancer cytoplasm contributes to increased glycolysis and an increase flux into glycolysis metabolic pathways, including the pentose phosphate pathway (PPP). The alteration of metabolites in PPP indicated a Warburg effect. Ornithine and arginine are the intermediate metabolites of urea cycle and the precursors of polyamines. The polyamines such as spermine, spermidine, and putrescine are known salivary biomarkers in OSCC. The obstruction in polyamine synthesis is associated with cancer cell proliferation [94,126,127,130,131,134].

The biochemical pathways like alanine, aspartate, the glutamate metabolism pathway, and the arginine–proline metabolism pathway are associated with mutant p53 status, which is frequent in OSCC [147]. Amino acid metabolism affects cancer, and is associated with increased glycolysis during cell proliferation in cancer tissues [142]. In one study, it was hypothesized that OSCC tumor cells absorb amino acid mainly glycine from the salivary extracellular space, and then tumor cells actively synthesize glycine in the mitochondria. In mitochondria, it forms one-carbon units for nucleotide synthesis, which further support tumor progression [134]. Taurine is an amino acid predominantly found in muscles and the brain, and it functions as antioxidant, anti-inflammatory and osmoregulation. Tryptophan is also a potential biomarker required for protein synthesis in oral cancer. It is a precursor to various bioactive metabolites that are involved in neurotransmission, antioxidant, energetic pathways, and genomic stability [148]. The abnormal choline and sphingolipid metabolism imply cell proliferation dysregulation [149]. Cholesterol metabolism contributes to bile acid and steroid hormone synthesis but, when altered, it promotes tumorigenesis and cancer progression by modulating signals [150].

## 5. Discussion

**Figure 2:**
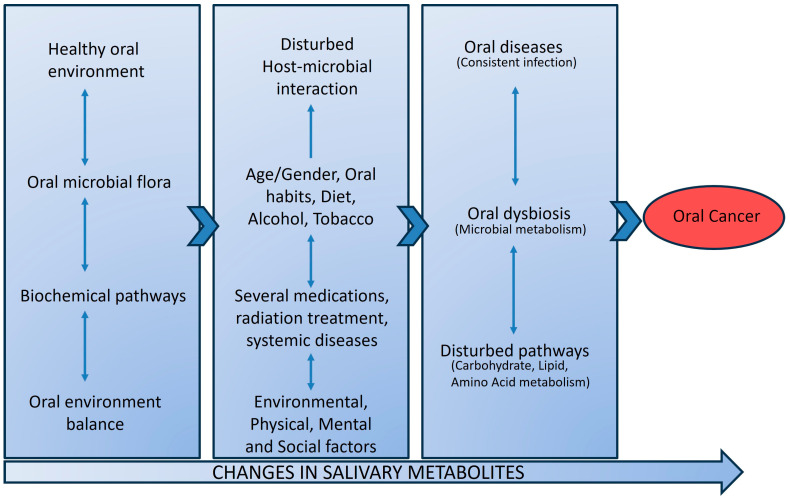
Pathologic model presenting oral dysbiosis and microbial metabolite with disturbed pathways influencing oral cancer development. In disease-free conditions, the oral microbial flora and their related biochemical pathways are in harmony to maintain a healthy oral environment. Disturbances in the host and oral microbiome can result in oral dysbiosis with disturbed biochemical pathways. Continued oral infection and oral dysbiosis affect the carbohydrate, amino acid, and lipid metabolism. The bacterial end-product in saliva as salivary metabolites can have carcinogenic effects, resulting in oral cancer.

It is unlikely that OSCC can be detected using a single biomarker with high specificity and sensitivity, because OSCCs are multifactorial in nature and possess heterogeneity in oncogenic pathways. Another major factor in the OSCC research is different microbial populations in different anatomical regions and the composition of oral microbiome communities varies by the saliva and distinct sites of the mouth. Alterations in microbial diversity, known risk factors (alcohol and tobacco), and unknown factors could actively contribute to OSCC tumorigenesis. Microbes can produce carcinogenic metabolite products that may influence carcinogenesis by altering host cell proliferation and death, disturbing immune system function, and influencing metabolism within a host. Such pathologic models for oral dysbiosis and microbial metabolites production influencing the oral carcinogenesis are presented in Figure 2.

In OSCC, the most differential genera observed increased Fusobacterium and decreased Streptococcus. Such a shift in the bacterial genera favors a more inflammatory state of oral epithelium. Moreover, *Streptococcus* spp. have been shown to impair *Fusobacterium nucleatum*-induced inflammation in oral epithelial cells [151]. The complex oral biofilm is initially colonized by *Streptococcus* on oral epithelium and later *Fusobacterium* spp. prompt coaggregation with other bacterial genera. The formation of oral biofilm promotes the invasiveness of *Fusobacterium nucleatum* into oral mucosa [152]. *Fusobacterium nucleatum* presence was demonstrated to protect tumor cells from immune cell attack and accelerate OSCC development via Toll-like receptors present in oral epithelium [153,154]. *Streptococcus anginosus*, *Veillonella parvula*, *Porphyromonas endodontalis*, and *Peptostreptococcus anaerobius* are considered as oncobacteria. These oncobacteria can contribute to OSCC development by increasing inflammation via increased expression of inflammatory cytokines [110]. These findings support an oncogenic role of oral dysbiosis microbial environment in oral cancer development.

The involvement of oral bacteria in oral cancer development is complex, and it may include chronic inflammation, alteration in cell homeostasis, the release of harmful substances, and compromised host response [95]. Oral microbiomes and their released products in the oral cavity have ability to activate fibroblasts and immune cells and produce reactive oxygen species (ROS) that trigger DNA damage in epithelial cells [155]. Oral microbial metabolites (hydrogen sulfide, ammonia, and fatty acids) may directly target DNA and elicit mutations. Also, it was found that microbial metabolism is associated with various biosynthetic pathways (Table 3). Most of the pathogenic periodontal bacteria are increased in OSCC. The host proteins are metabolized or fermented into sulfides and nitrosamines by *Firmicutes* and *Bacteroides*, thereby potentiating cell mutations [156].

The saliva samples of OSCC patients are enriched with *Capnocytophaga gingivalis*, *Prevotella melaninogenica*, *Sreptococcus mitis*, *Fusobacterium periodonticum*, *Prevotella tannerae*, *Neisseria*, *Lactobacillus*, *Bacteroides*, and *Prevotella intermedia* (Table 2). These are mobile periodontal pathogens associated with OSCC, and with extra-oral infection and inflammation. The periodontal bacteria can produce genotoxic and mutagenic agent hydrogen sulfide (H2S) in the gingival pockets that can induce chronic inflammation and cell proliferation, migration, invasion, and tumor angiogenesis [157]. The presence of periodontal bacteria is also observed in the OSCC tissue samples. It is suggested that the hypoxic tumor environment, the reduced host immunity, and the purines production in tumor necrotic tissue enable the attraction of bacteria into it. Fusobacterium nucleatum is observed both in saliva and tissue samples of OSCC, implicating the progression of OSCC. It induces oral epithelial cell proliferation through activation of kinases and binding to E-cadherin and, thereby, activating Wnt/β-catenin pathway. β-catenin signaling results in the activation of genes that control cell survival and proliferation [121].

With the deep-learning and machine learning methods, salivary metabolomics of periodontitis and oral cancer has shown leucine, aspartic acid, lactic acid, ornithine, tryptophan, glutamine, phenylalanine, taurine, glutathione, acetic acid, mannose, 24,25-dihydroxyvitamin D3, glutamic acid, glucose, epi-androsterone and 5,6 IP4 as common metabolites. Genes like Akt kinase (AKT), Phosphoinositide 3-kinase (PI3K), Extracellular signal-regulated protein kinases 1 and 2 (ERK1/2), p38 mitogen activated protein kinase (P38 MAPK) and Protein kinase C (PKC) are known cancer-related genes, and some of them are also involved in periodontitis [158]. Lactic acid metabolite is the result of carbohydrate fermentation due to the poor oral hygiene, and it is upregulated both in periodontitis [159] and OSCC [123,141]. Acetone originates from breakdown of acetoacetate and α-hydroxybutyrate, accompanying fatty acid degradation, glycolysis, and pyruvate metabolism. Glycerol is a metabolite that can originate from glucose, proteins, pyruvate, triacylglycerols, and other metabolic pathways. Glycerol-3-phosphate, which is the product of glycerol phosphorylation by glycerol kinase, is elevated both in periodontitis [160] and OSCC [141]. Another common metabolite is methanol, an endogenous metabolite that might regulate mammalian gene activity [161]. The source of methanol in humans is not well known, but it can be formed by the transformation of S-adenosyl methionine to methanol. The lower level of methanol can be related to its use as a carbon or energy source for bacteria associated with periodontitis [159] and carcinogenesis [129].

It is evident from the above findings that oral microbiome has the potential to induce chronic inflammation, and can produce carcinogenic metabolites that could promote oral diseases, including OSCC. The promising adjunct therapies to promote the growth of beneficial bacteria and reduce pathogenic bacteria are prebiotics and probiotics [162]. Prebiotics and probiotics could be potential therapeutic interventions for oral microbial balance and improving overall oral and systemic health. Also, for clinicians and other practitioners, it is important to understand the crucial role of the oral microbiome and its metabolites in oral diseases, as it could further assist in targeted therapies and personalized medicine in the clinical scenario.

## 6. Conclusions

This review supports the interplay between oral microbiome and its released metabolites with OSCC tumorigenesis. Several commensal bacteria transform to pathogenic bacteria under favorable conditions in the oral cavity and influence the progression of oral cancer. Hence, oral dysbiosis is a risk factor in oral cancer development through different mechanisms, and thus it positively or negatively influences the outcome response to cancer therapy. This review provides insights into oral bacterial species, oral dysbiosis or microbial metabolites of potential importance to be investigated on saliva samples in patients with OSCC. These dysregulated metabolite markers are diagnostic indicators of OSCC and help in evaluating the potential therapeutic outcome of OSCC. Salivary metabolomic is an important noninvasive opportunity to investigate metabolic pathways associated with oral microbes and tumorigenesis of OSCC.

## Figures and Tables

**Table 1:** Oral microbial metabolites produced in periodontal tissue, and their function in oral biofilm and on the host.

Oral Microbiome	Breakdown Compounds	Metabolites	Oral Biofilm	Host Response	Ref
*Actinomyces* spp. *Bacteroides* spp. *Corynebacteria* spp. *Eubacterium* spp. *Fusobacterium* spp. *Haemophilus* spp. *Megasphaera* spp. *Neisseria* spp. *Propionibacterium* *Prevotella* spp. *Porphyromonas* spp. *Rothia* spp.	Carbohydrates, proteins, amino acids	Short-chain fatty Acids (SCFAs): Acetate Butyrate Formate Propionate	Antibacterial activity	Pro-inflammatory Anti-inflammatory Chemoattractant Gut-Brain Interaction	[45,46,47,48,49,50,51]
*Porphyromonas* *gingivalis* *Prevotella intermedia*	Carbohydrates, proteins, amino acids	Organic acids: carboxylic, caproic, Isocaproic, succinate, phenylacetic acid	Antibacterial activity	Chemoattractant	[52]
*Fusobacterium* spp. *Porphyromonas* spp. *Prevotella* spp. *Tannerella* spp. *Treponema* spp. *Lactobacillus* spp. *Peptostreptococcus* spp. *Helicobacter pylori* *Campylobacter* *ureolyticus* *Haemophilus* *parainfluenzae* *Streptococcus* spp. *Actinomyces* spp. *Staphylococcus* spp. *Rothia dentocariosa*	Proteins/amino acids: Arginine Lysine Methionine Cysteine Cystine Tryptophan Urea	Ammonia	Antibiotic resistance Inhibits neutrophil function	Toxic and impaired function of neutrophils	[42,45,52,53]
*Streptococcus* spp. *Lactobacillus* spp.	Carbohydrates Proteins/amino acids: Arginine Methionine Cysteine Urea	Carbon dioxide	Stimulus for the growth of most anaerobes	Toxic	[54,55,56,57,58]
*Campylobacter* spp.	Carbohydrates Proteins/amino acids	Hydrogen gas	Bacterial survival and growth	Anti-inflammatory	
*Streptococcus mitis*	Heme	Carbon monoxide	Antimicrobial	Gasotransmitter	
*Veillonella* spp. *Rothia* spp. *Actinomyces* spp.	Nitrate	Nitric oxide	Bactericidal Increased resistance to antibiotics Antibacterial activity Increased biofilm dispersal	Gasotransmitter	
*Fusobacterium* spp. *Parvimonas micra* *Porphyromonas* spp. *Prevotella intermedia* *Treponema denticola* *Streptococcus* *anginosus* *Desulfobacter* spp. *Desulfovibrio* spp. *Desulfomicrobium* *orale*	Cysteine Sulfate	Hydrogen sulfide	Harmful in high concentrations Increased resistance to antibiotics Increased resistance to immune-mediated killing, Protection from oxidative stress	Toxic at high concentrations Pro-inflammatory Anti-inflammatory Gasotransmitter	[43,59,60]
*Fusobacterium* spp.	Methionine	Methyl mercaptan	Altered biofilm composition	Decrease collagen synthesis Pro-inflammatory	[59,61]
*Campylobacter* spp. *Archaea* *Methanobrevibacter*	Hydrogen gas Carbon dioxide Acetate Methylamine	Methane	Not known	Associated with severe colonic diseases	[62]
*Streptococcus* spp.	Oxygen Pyruvate	Hydrogen peroxide	Regulatory function	Inhibition of inflammasomes	[63,64]
*Fusobacterium* spp. *Lactobacillus* spp. *Prevotella* spp. *Porphyromonas* spp. *Streptococcus* spp. *Treponema denticola*	Tryptophan Lysine Ornithine Arginine	Amines-Indole Skatole Cadaverine Putrescine Spermine Spermidine	Increased resistance to antibiotics, Formation on biofilm, cell metabolism, cell differentiation, plasmid stability, drug resistance, signaling	Bacterial virulence Toxic Cell physiology	[65,66,67,68,69]

**Table 2:** The oral microbiome, identified in the saliva samples of OSCC patients.

S.NO	Sample (OSCC/Controls)	Method	Oral Microbiome	Ref.
1	USWS (45/45)	DNA-DNA hybridization	*Capnocytophaga gingivalis*, *Prevotella melaninogenica* and *Streptococcus mitis*,	[98]
2	SWS (3/2)	16S rRNA PCR	*Firmicutes* and *Bacteroidetes*	[99]
3	USWS (6/25)	16S rRNA PCR	*Firmicutes*, *Streptococcus* and *Prevotella*, *Lactobacillus*, *Neisseria*, *Enterobacteriaceae*, *Oribacterium*, *Bacteroidetes* and *Proteobacteria*	[100]
4	USWS (125/127)	16S rRNA PCR	*Bacillus*, *Enterococcus*, *Parvimonas*, *Peptostreptococcus*, and *Slackia*	[101]
5	USWS (6/25)	16S rRNA NGS	*Lactobacillus gasseri*, *Lactobacillus johnsonii*, and *Fusobacterium_nucleatum*	[102]
6	USWS (14/16)	16S rRNA PCR	*Prevotella*, *Neisseria*, *Rothia*, *Streptococcus* and *Veillonella*	[103]
7	USWS (88/90)	16S rRNA PCR	*Prevotella tannerae*, *Fusobacterium nucleatum* and *Prevotella intermedia*	[104]
8	USWS (6/4)	16S rRNA PCR	*Bacteroidetes* and *genus Solobacterium*	[105]
9	SWS (60/80)	16S rRNA PCR	*Peptostreptococcus*, *Fusobacterium*, *Alloprevotella*, and *Capnocytophaga*	[106]
10	USWS (10/15)	16S rRNA PCR	*Fusobacterium*, *Peptostreptococcus*, and *Prevotella*, *Streptococcus*, *Neisseria*, and *Haemophilus*	[107]
11	USWS (31/23)	16S rRNA PCR	*Megasphaera*, *unclassified Enterobacteriae*, *Salmonella* and *Prevotella*	[108]
12	USWS (56/64)	16S rRNA PCR	*Capnocytophaga* and *Neisseria*	[109]
13	USWS (25/24)	16S rRNA PCR	*Prevotella*, *Fusobacterium*, *Porphyromonas*, *Streptococcus*, *Capnocytophaga*, *Haemophilus*, *Neisseria*, *Rothia*, and *Veillonella*	[110]
14	USWS (59/32)	16S rRNA NGS	*Candida*, *Malassezia*, *Saccharomyces*, *Aspergillus*, and *Cyberlindnera*	[111]
15	USWS (47/48)	16S rRNA PCR	*Actinobacteria*, *Fusobacterium*, *Moraxella*, *Bacillus*, and *Veillonella*	[112]
16	USWS (23/18)	16S rRNA PCR	*Prevotella*	[113]
17	USWS (16/8)	16S rRNA PCR	*Rothia*, *Veillonella*, *Staphylococcus*, *Centipeda*, *Dialister*, *Gemella*, *Granulicatella*, *Firmicutes* and *Actinobacteria*	[114]
18	USWS (24/7)	16S rRNA PCR	*Prevotella*, *Chlamydia*, *Tissierellia*, *Calothrix*, *Leotiomycetes*, *Firmicutes* and *Zetaproteobacteria*	[115]
19	USWS (99/101)	16S rRNA PCR	*Streptococcus anginosus*, *Abiotrophia defectiva*, and *Fusobacterium nucleatum*	[116]

S.NO—serial number, OSCC—oral squamous cell carcinoma, USWS—unstimulated whole saliva, SWS—stimulated whole saliva, DNA—deoxyribose nucleic acid, rRNA—ribosomal ribose nucleic acid, PCR—polymerase chain reaction.

**Table 3:** Oral microbial metabolites and the affected biochemical metabolic pathway in OSCC.

Carbohydrates	Amino Acids	Organic Acids	Fatty Acids	Lipids	Amines	Amides	Metabolic Pathway	Ref.
N-Acetyl-D-glucosamine	N-Acetyl-L-phenylalanine, D-Alanyl-D-alanine, Palmitoyl-L-carnitine, N-Glycyl-L-proline, L-Carnitine	L-Pipecolic acid		phosphorylcholine	Deoxyguanosine		Glycolysis, Phospholipid and choline metabolism, Fatty acid oxidation, Oxidative stress biosynthesis	[124]
	5,5-diethylpentadecane, L-proline	decanedioic acid, 2-methyloctacosane, Eicosane, Octane, 3,5-dimethyl, pentadecane, hentriacontane, nonadecane, oxalic acid, 6-phenylundecanea, 2-furancarboxamide, 2-isopropyl-5-methyl-1-heptanol, pentanoic acid, docosane					Amino acid metabolism Organic acid metabolism	[125]
N-acetylglucosamine	proline, carnitine, 5-hydroxylysine, 3-methylhistidine				adenosine, inosine			[126]
maltose, dihydroxyacetone phosphate, galacturonic acid, ribose 5-phosphate, lactose	methionine, inosine, uracil, o-phospho-serine, pantothenic acid, leucine			malic acid, protocatechuic acid, 2-ketoglutaric acid, catechol, 2-ketoadipic acid, margaric acid, palmitic acid, maleic acid	indole-3-acetic acid, spermidine	urea	Malate-Asparate shuttle pathway, Warburg effect pathway, Beta-alanine pathway	[127]
	histidine, tyrosine, glycine, glutamic acid, aspartic acid, tryptophan, lysine, methionine, gamma-aminobutyric acid (GABA), urocanate, 2-isopropaylate, 2-aminobutyric acids		butyrate				TCA cycle, Tryptophan metabolism	[128]
fucose	taurine, glycine, aspartate, cisaconitate, glycine	methanol	propionate, isobutyrate, acetoacetate	choline	trimethylamine N-oxide		Tryptophan and Nicotinamide pathway	[129]
N-acetylglucosamine	creatinine, 5-aminovalerate, pipecolate, gamma-butyrobetaine, 2′-deoxyinsine, N-acetylhistidine, o-acetylcarnitine	N-acetylputrescine, indole-3-acetate		ethanolamine phosphate	trimethylamine N-oxide, putrescine, N1-acetylspermine		Methane, Purine, Glutathione, lysine, sphingolipid, Arginine, proline, Glycerophospholipid metabolism	[94]
glucose	cadaverine, serine	5-aminopentoate, hippuric acid		phosphocholine, adrenic acid	putrescine, thymidine, adenosine		Amino acid biosynthesis, Arginine and proline pathway, histidine, lysine pathway	[130]
N-acetylglucosamine-1-phosphate, ribose 5-phosphate (R5P)	carnitine arginine	o-hydroxybenzoate		ornithine			Pentose–phosphate pathway	[131]
3-heptanone, pentanone, butyrolactone		1,3-butanediol, 1,2-pentanediol, 1-hexadecanol, ethanol, 2-phenol, 1-octanol, benzyl alcohol		hexadecanoic acid, undecane			Fatty acid biosynthesis	[132]
d-glycerate-2-phosphate, 4-nitroquinoline-1-oxide, inositol 1,3,4-triphosphate, neuraminic acid	1-methyl histidine, 2-oxoarginine, norcocaine nitroxide, p-chlorphenylalanine, N-(3-Indolylacetyl)-l-isoleucine, l- homocysteic acid, ubiquinone	S-ureidoglycolic acid, d-urobilinogen		estrone-3-glucuronide, sphinganine-1 phosphate, tetradecanedioic acid, 1-hexadecyl hexadecanoate, estradiol valerate	pseudouridine		Amino acid, Carbohydrate, Estrogen, Spingolipid metabolism, Oxidative stress, Neucleotide biosynthesis pathway, Electron transport	[133]
	glycine, proline, citrulline			ornithine			TCA cycle, Threonine, Arginine and proline pathway	[134]
fucose	Proline	1,2 propanediol					Carbohydrate and Amino acid metabolism	[135]
		1,4-dichlorobenzene, 1,2-decanediol, 2,5-Bis1, 1-dimethylethylphenol, E-3-decen-2-ol, 2,4-dimethyl-1-heptene, 1-chloro-2-propanol, 1-chloro-2-butanol, 2-propenoic acid, 2,3,3-trimethylpentane, ethanol, 1,2,3,4-tetrachlorobutane	propanoic acid (ethyl ester), acetic acid, propanoic acid, ethyl acetate				Amino acid metabolism, Propanoate metabolism, Glycolysis, Pyruvate, Sulphur and Taurine metabolism, Nicotinate pathway, Ketone bodies pathway	[136]
3-phenyllactic acid	2-hydroxy-4-methylvaleric acid, valine, leucine, butyrobetaine, isoleucine, tryptophan, 3-phenylpropionic acid, cadaverine, N6,N6,N6-trimethyllysine, taurine, alanine	p-hydroxyphenylacetic acid, hexanoic acid, octanoic acid, terephthalic acid, glycolic acid, heptanoic acid	3-propionic acid, butyric acid, 2-oxoisovaleric acid	choline		urea	Urea cycle	[137]
3-phosphoglyceric acid	pipecolate. methionine, S-adenosylmethionine, tryptophan, valine, hypoxanthine, glycylglycine, taurine, cadaverine			choline	spermidine, 2-aminobenzamide, trimethylamine N-oxide, guanine, guanosine, threonine		Polyamine synthesis	[138]
	L-phenylalanine and L-leucine						TCA cycle, Fat metabolism	[139]
	betaine, L-carnitine	pipecolinic acid		choline			Lipid, Lysine and Fatty acid metabolism	[140]
	N-nonanoylglycine, hexanoylcarnitine, carnitine, 4-hydroxy-L-glutamic acid, acetylphenylalanine, S-carboxymethyl-L-cystein	lactic acid, hydroxyphenynactic acid, succinic acid		ornithine, propionylcholine, spihingarine, phytosphingosine	hydroxymethyluracil		Amino acid metabolism, Fatty acid and carbohydrate metabolism, TCA cycle, Urea cycle	[141]
	alanine, 3-indolepropionic acid, valine, proline, isoleucine, leucine, proline, threonine, phenylalanine, γ-aminobutyric acid	lactic acid,		n-eicosanoic acid, n-tetradecanoic acid			Krebs cycle	[123]
	pyrroline hydroxycarboxylic acid, leucine plus isoleucine, tryptophan, valine, threonine, histidine, pipecolic acid, glutamic acid, carnitine, alanine, taurine, C_4_H_9_N and C_8_H_9_N, phenylalanine betaine, serine, tyrosine, glutamine, beta-alanine, cadaverine, C_5_H_14_N_5,_ C_4_H_5_N_2_O_11_P		alpha-aminobutyric acid	choline	piperideine, C_6_H_6_N_2_O_2_,		Phospholipid pathway	[142]
	Vitamin B and C						Lipid peroxidation	[143]

## Data Availability

All data presented in this study is presented in the articles menthioned in References.

## References

[1] Sung H., Ferlay J., Siegel R.L., Laversanne M., Soerjomataram I., Jemal A., Bray F. (2021). Global cancer statistics 2020: GLOBOCAN estimates of incidence and mortality worldwide for 36 cancers in 185 countries. CA A Cancer J. Clin..

[2] Kurago Z., Loveless J. (2021). Microbial colonization and inflammation as potential contributors to the lack of therapeutic success in oral squamous cell carcinoma. Front. Oral Health.

[3] Bacali C., Vulturar R., Buduru S., Cozma A., Fodor A., Chiș A., Lucaciu O., Damian L., Moldovan M.L. (2022). Oral Microbiome: Getting to Know and Befriend Neighbors, a Biological Approach. Biomedicines.

[4] HOMD Human Oral Microbiome Database. http://www.homd.org/.

[5] Hyvärinen E., Savolainen M., Mikkonen J.J.W., Kullaa A.M. (2021). Salivary Metabolomics for Diagnosis and Monitoring Diseases: Challenges and Possibilities. Metabolites.

[6] Metsäniitty M., Hasnat S., Salo T., Salem A. (2021). Oral Microbiota—A New Frontier in the Pathogenesis and Management of Head and Neck Cancers. Cancers.

[7] Tuominen H., Rautava J. (2021). Oral Microbiota and Cancer Development. Pathobiology.

[8] Zürcher C., Humpel C. (2023). Saliva: A challenging human fluid to diagnose brain disorders with a focus on Alzheimer’s disease. Neural Regen. Res..

[9] Barnes V.M., Kennedy A.D., Panagakos F., Devizio W., Trivedi H.M., Jönsson T., Guo L., Cervi S., Scannapieco F.A. (2014). Global metabolomic analysis of human saliva and plasma from healthy and diabetic subjects, with and without periodontal disease. PLoS ONE.

[10] Song M., Bai H., Zhang P., Zhou X., Ying B. (2023). Promising applications of human-derived saliva biomarker testing in clinical diagnostics. Int. J. Oral Sci..

[11] Hyvärinen E., Kashyap B., Kullaa A.M. (2023). Oral Sources of Salivary Metabolites. Metabolites.

[12] Nijakowski K., Gruszczyński D., Kopała D., Surdacka A. (2022). Salivary Metabolomics for Oral Squamous Cell Carcinoma Diagnosis: A Systematic Review. Metabolites.

[13] Abisado R.G., Benomar S., Klaus J.R., Dandekar A.A., Chandler J.R. (2018). Bacterial Quorum Sensing and Microbial Community Interactions. mBio.

[14] Li K., Bihan M., Methé B.A. (2013). Analyses of the stability and core taxonomic memberships of the human microbiome. PLoS ONE.

[15] Sharma N., Bhatia S., Sodhi A.S., Batra N. (2018). Oral microbiome and health. AIMS Microbiol..

[16] Aas J.A., Paster B.J., Stokes L.N., Olsen I., Dewhirst F.E. (2005). Defining the normal bacterial flora of the oral cavity. J. Clin. Microbiol..

[17] Dawes C. (2003). Estimates, from salivary analyses, of the turnover time of the oral mucosal epithelium in humans and the number of bacteria in an edentulous mouth. Arch. Oral Biol..

[18] Itzek A., Gillen C.M., Fulde M., Friedrichs C., Rodloff A.C., Chhatwal G.S., Nitsche-Schmitz D.P. (2010). Contribution of plasminogen activation towards the pathogenic potential of oral streptococci. PLoS ONE.

[19] Leonov G.E., Varaeva Y.R., Livantsova E.N., Starodubova A.V. (2023). The Complicated Relationship of Short-Chain Fatty Acids and Oral Microbiome: A Narrative Review. Biomedicines.

[20] Tonelli A., Lumngwena E.N., Ntusi N.A.B. (2023). The oral microbiome in the pathophysiology of cardiovascular disease. Nat. Rev. Cardiol..

[21] Sureda A., Daglia M., Argüelles Castilla S., Sanadgol N., Fazel Nabavi S., Khan H., Belwal T., Jeandet P., Marchese A., Pistollato F. (2020). Oral microbiota and Alzheimer’s disease: Do all roads lead to Rome?. Pharmacol. Res..

[22] Chu X.J., Cao N.W., Zhou H.Y., Meng X., Guo B., Zhang H.Y., Li B.Z. (2021). The oral and gut microbiome in rheumatoid arthritis patients: A systematic review. Rheumatology.

[23] Jang H., Patoine A., Wu T.T., Castillo D.A., Xiao J. (2021). Oral microflora and pregnancy: A systematic review and meta-analysis. Sci. Rep..

[24] Read E., Curtis M.A., Neves J.F. (2021). The role of oral bacteria in inflammatory bowel disease. Nat. Rev. Gastroenterol. Hepatol..

[25] Wang J., Gao Y., Zhao F. (2016). Phage-bacteria interaction network in human oral microbiome. Environ. Microbiol..

[26] Wahida A., Ritter K., Horz H.P. (2016). The Janus-Face of Bacteriophages across Human Body Habitats. PLoS Pathog..

[27] Pride D.T., Salzman J., Haynes M., Rohwer F., Davis-Long C., White R.A., Loomer P., Armitage G.C., Relman D.A. (2012). Evidence of a robust resident bacteriophage population revealed through analysis of the human salivary virome. ISME J..

[28] Ghannoum M.A., Jurevic R.J., Mukherjee P.K., Cui F., Sikaroodi M., Naqvi A., Gillevet P.M. (2010). Characterization of the oral fungal microbiome (mycobiome) in healthy individuals. PLoS Pathog..

[29] Wu Y.M., Yan J., Ojcius D.M., Chen L.L., Gu Z.Y., Pan J.P. (2007). Correlation between infections with different genotypes of human cytomegalovirus and Epstein-Barr virus in subgingival samples and periodontal status of patients. J. Clin. Microbiol..

[30] Betz S.J. (2019). HPV-Related Papillary Lesions of the Oral Mucosa: A Review. Head Neck Pathol..

[31] Jakubovics N.S., Kolenbrander P.E. (2010). The road to ruin: The formation of disease-associated oral biofilms. Oral Dis..

[32] Kreft J.U. (2004). Biofilms promote altruism. Microbiology.

[33] García-Curiel L., Del Rocío López-Cuellar M., Rodríguez-Hernández A.I., Chavarría-Hernández N. (2021). Toward understanding the signals of bacteriocin production by *Streptococcus* spp. and their importance in current applications. World J. Microbiol. Biotechnol..

[34] Jakubovics N.S., Gill S.R., Vickerman M.M., Kolenbrander P.E. (2008). Role of hydrogen peroxide in competition and cooperation between *Streptococcus gordonii* and *Actinomyces naeslundii*. FEMS Microbiol. Ecol..

[35] Edwards A.M., Grossman T.J., Rudney J.D. (2006). *Fusobacterium nucleatum* transports noninvasive *Streptococcus cristatus* into human epithelial cells. Infect. Immun..

[36] Kuramitsu H.K., He X., Lux R., Anderson M.H., Shi W. (2007). Interspecies interactions within oral microbial communities. Microbiol. Mol. Biol. Rev..

[37] Bowen W.H., Burne R.A., Wu H., Koo H. (2018). Oral Biofilms: Pathogens, Matrix, and Polymicrobial Interactions in Microenvironments. Trends Microbiol..

[38] Lamont R.J., Koo H., Hajishengallis G. (2018). The oral microbiota: Dynamic communities and host interactions. Nat. Rev. Microbiol..

[39] Sintim H.O., Gürsoy U.K. (2016). Biofilms as “Connectors” for Oral and Systems Medicine: A New Opportunity for Biomarkers, Molecular Targets, and Bacterial Eradication. OMICS.

[40] Aruni A.W., Dou Y., Mishra A., Fletcher H.M. (2015). The Biofilm Community-Rebels with a Cause. Curr. Oral Health Rep..

[41] Curtis M.A., Griffiths G.S., Price S.J., Coulthurst S.K., Johnson N.W. (1988). The total protein concentration of gingival crevicular fluid. Variation with sampling time and gingival inflammation. J. Clin. Periodontol..

[42] Bernier S.P., Létoffé S., Delepierre M., Ghigo J.M. (2011). Biogenic ammonia modifies antibiotic resistance at a distance in physically separated bacteria. Mol. Microbiol..

[43] Shatalin K., Shatalina E., Mironov A., Nudler E. (2011). H2S: A universal defense against antibiotics in bacteria. Science.

[44] Gusarov I., Shatalin K., Starodubtseva M., Nudler E. (2009). Endogenous nitric oxide protects bacteria against a wide spectrum of antibiotics. Science.

[45] Uematsu H., Sato N., Hossain M.Z., Ikeda T., Hoshino E. (2003). Degradation of arginine and other amino acids by butyrate-producing asaccharolytic anaerobic Gram-positive rods in periodontal pockets. Arch. Oral Biol..

[46] Ochiai K., Kurita-Ochiai T. (2009). Effects of butyric acid on the periodontal tissue. Jpn. Dent. Sci. Rev..

[47] Huang C.B., Alimova Y., Myers T.M., Ebersole J.L. (2011). Short- and medium-chain fatty acids exhibit antimicrobial activity for oral microorganisms. Arch. Oral Biol..

[48] Dahlstrand Rudin A., Khamzeh A., Venkatakrishnan V., Basic A., Christenson K., Bylund J. (2021). Short chain fatty acids released by *Fusobacterium nucleatum* are neutrophil chemoattractants acting via free fatty acid receptor 2 (FFAR2). Cell Microbiol..

[49] Dahlstrand Rudin A., Khamzeh A., Venkatakrishnan V., Persson T., Gabl M., Savolainen O., Forsman H., Dahlgren C., Christenson K., Bylund J. (2021). *Porphyromonas gingivalis* Produce Neutrophil Specific Chemoattractants Including Short Chain Fatty Acids. Front. Cell. Infect. Microbiol..

[50] Guan X., Li W., Meng H. (2021). A double-edged sword: Role of butyrate in the oral cavity and the gut. Mol. Oral Microbiol..

[51] Silva Y.P., Bernardi A., Frozza R.L. (2020). The Role of Short-Chain Fatty Acids From Gut Microbiota in Gut-Brain Communication. Front. Endocrinol..

[52] Takahashi N., Saito K., Schachtele C.F., Yamada T. (1997). Acid tolerance and acid-neutralizing activity of *Porphyromonas gingivalis*, *Prevotella intermedia* and *Fusobacterium nucleatum*. Oral Microbiol. Immunol..

[53] Niederman R., Brunkhorst B., Smith S., Weinreb R.N., Ryder M.I. (1990). Ammonia as a potential mediator of adult human periodontal infection: Inhibition of neutrophil function. Arch. Oral Biol..

[54] Siracusa R., Voltarelli V.A., Salinaro A.T., Modafferi S., Cuzzocrea S., Calabrese E.J., Di Paola R., Otterbein L.E., Calabrese V. (2022). NO, CO and H2S: A trinacrium of bioactive gases in the brain. Biochem. Pharmacol..

[55] Audrain B., Farag M.A., Ryu C.M., Ghigo J.M. (2015). Role of bacterial volatile compounds in bacterial biology. FEMS Microbiol. Rev..

[56] Kuboniwa M., Sakanaka A., Hashino E., Bamba T., Fukusaki E., Amano A. (2016). Prediction of Periodontal Inflammation via Metabolic Profiling of Saliva. J. Dent. Res..

[57] Nguyen D., Nguyen T.K., Rice S.A., Boyer C. (2015). CO-Releasing Polymers Exert Antimicrobial Activity. Biomacromol..

[58] Jaffe F.A. (1997). Pathogenicity of carbon monoxide. Am. J. Forensic Med. Pathol..

[59] Persson S., Edlund M.B., Claesson R., Carlsson J. (1990). The formation of hydrogen sulfide and methyl mercaptan by oral bacteria. Oral Microbiol. Immunol..

[60] Dilek N., Papapetropoulos A., Toliver-Kinsky T., Szabo C. (2020). Hydrogen sulfide: An endogenous regulator of the immune system. Pharmacol. Res..

[61] Johnson P., Yaegaki K., Tonzetich J. (1996). Effect of methyl mercaptan on synthesis and degradation of collagen. J. Periodontal. Res..

[62] Sogodogo E., Drancourt M., Grine G. (2019). Methanogens as emerging pathogens in anaerobic abscesses. Eur. J. Clin. Microbiol. Infect. Dis..

[63] Zhu L., Kreth J. (2012). The role of hydrogen peroxide in environmental adaptation of oral microbial communities. Oxid. Med. Cell Longev..

[64] Erttmann S.F., Gekara N.O. (2019). Hydrogen peroxide release by bacteria suppresses inflammasome-dependent innate immunity. Nat. Commun..

[65] Codipilly D., Kleinberg I. (2008). Generation of indole/skatole during malodor formation in the salivary sediment model system and initial examination of the oral bacteria involved. J. Breath Res..

[66] Lee J.H., Lee J. (2010). Indole as an intercellular signal in microbial communities. FEMS Microbiol. Rev..

[67] Inaba T., Obana N., Habe H., Nomura N. (2020). Biofilm Formation by *Streptococcus mutans* is Enhanced by Indole via the Quorum Sensing Pathway. Microbes Environ..

[68] Amin M., Tang S., Shalamanova L., Taylor R.L., Wylie S., Abdullah B.M., Whitehead K.A. (2021). Polyamine biomarkers as indicators of human disease. Biomarkers.

[69] Wójcik W., Łukasiewicz M., Puppel K. (2021). Biogenic amines: Formation, action and toxicity—A review. J. Sci. Food Agric..

[70] Kilian M. (2018). The oral microbiome—Friend or foe?. Eur. J. Oral Sci..

[71] Zhou J., Jiang N., Wang S., Hu X., Jiao K., He X., Li Z., Wang J. (2016). Exploration of Human Salivary Microbiomes—Insights into the Novel Characteristics of Microbial Community Structure in Caries and Caries-Free Subjects. PLoS ONE.

[72] Wang Y., Zhang J., Chen X., Jiang W., Wang S., Xu L., Tu Y., Zheng P., Wang Y., Lin X. (2017). Profiling of Oral Microbiota in Early Childhood Caries Using Single-Molecule Real-Time Sequencing. Front. Microbiol..

[73] van Houte J. (1994). Role of micro-organisms in caries etiology. J. Dent. Res..

[74] Schulz A., Lang R., Behr J., Hertel S., Reich M., Kümmerer K., Hannig M., Hannig C., Hofmann T. (2020). Targeted metabolomics of pellicle and saliva in children with different caries activity. Sci. Rep..

[75] Tomita Y., Miyake N., Yamanaka S. (2008). Lipids in human parotid saliva with regard to caries experience. J. Oleo Sci..

[76] Pereira J.L., Duarte D., Carneiro T.J., Ferreira S., Cunha B., Soares D., Costa A.L., Gil A.M. (2019). Saliva NMR metabolomics: Analytical issues in pediatric oral health research. Oral Dis..

[77] Li K., Wang J., Du N., Sun Y., Sun Q., Yin W., Li H., Meng L., Liu X. (2023). Salivary microbiome and metabolome analysis of severe early childhood caries. BMC Oral Health.

[78] Wang J., Qi J., Zhao H., He S., Zhang Y., Wei S., Zhao F. (2013). Metagenomic sequencing reveals microbiota and its functional potential associated with periodontal disease. Sci. Rep..

[79] Hoare A., Soto C., Rojas-Celis V., Bravo D. (2019). Chronic Inflammation as a Link between Periodontitis and Carcinogenesis. Mediators Inflamm..

[80] Citterio F., Romano F., Meoni G., Iaderosa G., Grossi S., Sobrero A., Dego F., Corana M., Berta G.N., Tenori L. (2020). Changes in the Salivary Metabolic Profile of Generalized Periodontitis Patients after Non-surgical Periodontal Therapy: A Metabolomic Analysis Using Nuclear Magnetic Resonance Spectroscopy. J. Clin. Med..

[81] Coussens L.M., Werb Z. (2002). Inflammation and cancer. Nature.

[82] Chen Y., Chen X., Yu H., Zhou H., Xu S. (2019). Oral Microbiota as Promising Diagnostic Biomarkers for Gastrointestinal Cancer: A Systematic Review. OncoTargets Ther..

[83] Mascitti M., Togni L., Troiano G., Caponio V.C.A., Gissi D.B., Montebugnoli L., Procaccini M., Lo Muzio L., Santarelli A. (2019). Beyond Head and Neck Cancer: The Relationship between Oral Microbiota and Tumour Development in Distant Organs. Front. Cell. Infect. Microbiol..

[84] Stasiewicz M., Karpiński T.M. (2022). The oral microbiota and its role in carcinogenesis. Semin. Cancer Biol..

[85] Lu R.F., Feng L., Gao X.J., Meng H.X., Feng X.H. (2013). [Relationship between volatile fatty acids and Porphyromonas gingivalis and Treponema denticola in gingival crevicular fluids of patients with aggressive periodontitis]. Beijing Da Xue Xue Bao Yi Xue Ban..

[86] Ojeda D., Huber M.A., Kerr A.R. (2020). Oral Potentially Malignant Disorders and Oral Cavity Cancer. Dermatol. Clin..

[87] Amer A., Galvin S., Healy C., Moran G.P. (2017). The microbiome of potentially malignant oral leukoplakia exhibits enrichment for *Fusobacterium*, *Leptotrichia*, *Campylobacter*, and *Rothia* species. Front. Microbiol..

[88] Pietrobon G., Tagliabue M., Stringa L.M., De Berardinis R., Chu F., Zocchi J., Carlotto E., Chiocca S., Ansarin M. (2021). Leukoplakia in the Oral Cavity and Oral Microbiota: A Comprehensive Review. Cancers.

[89] Amer A., Whelan A., Al-Hebshi N.N., Healy C.M., Moran G.P. (2020). Acetaldehyde production by *Rothia mucilaginosa* isolates from patients with oral leukoplakia. J. Oral Microbiol..

[90] Yokoyama S., Takeuchi K., Shibatam Y., Kageyama S., Matsumi R., Takeshita T., Yamashita Y. (2018). Characterization of oral microbiota and acetaldehyde production. J. Oral Microbiol..

[91] Li Y., Wang K., Zhang B., Tu Q., Yao Y., Cui B., Ren B., He J., Shen X., Van Nostrand J.D. (2019). Salivary mycobiome dysbiosis and its potential impact on bacteriome shifts and host immunity in oral lichen planus. Int. J. Oral Sci..

[92] He Y., Gong D., Shi C., Shao F., Shi J., Fei J. (2017). Dysbiosis of oral buccal mucosa microbiota in patients with oral lichen planus. Oral Dis..

[93] Du G.H., Wang Y.F., Chen J.J., Deng Y., Han X.Z., Tang G.Y. (2020). Potential association between *Fusobacterium nucleatum* enrichment on oral mucosal surface and oral lichen planus. Oral Dis..

[94] Ishikawa S., Sugimoto M., Edamatsu K., Sugano A., Kitabatake K., Iino M. (2020). Discrimination of oral squamous cell carcinoma from oral lichen planus by salivary metabolomics. Oral Dis..

[95] Chattopadhyay I., Verma M., Panda M. (2019). Role of Oral Microbiome Signatures in Diagnosis and Prognosis of Oral Cancer. Technol. Cancer Res. Treat..

[96] Clarridge J.E. (2004). Impact of 16S rRNA gene sequence analysis for identification of bacteria on clinical microbiology and infectious diseases. Clin. Microbiol. Rev..

[97] Woese C.R. (1987). Bacterial evolution. Microbiol. Rev..

[98] Mager D.L., Haffajee A.D., Devlin P.M., Norris C.M., Posner M.R., Goodson J.M. (2005). The salivary microbiota as a diagnostic indicator of oral cancer: A descriptive, non-randomized study of cancer-free and oral squamous cell carcinoma subjects. J. Transl. Med..

[99] Pushalkar S., Mane S.P., Ji X., Li Y., Evans C., Crasta O.R., Morse D., Meagher R., Singh A., Saxena D. (2011). Microbial diversity in saliva of oral squamous cell carcinoma. FEMS Immunol. Med. Microbiol..

[100] Guerrero-Preston R., Godoy-Vitorino F., Jedlicka A., Rodríguez-Hilario A., González H., Bondy J., Lawson F., Folawiyo O., Michailidi C., Dziedzic A. (2016). 16S rRNA amplicon sequencing identifies microbiota associated with oral cancer, human papilloma virus infection and surgical treatment. Oncotarget..

[101] Lee W.H., Chen H.M., Yang S.F., Liang C., Peng C.Y., Lin F.M., Tsai L.L., Wu B.C., Hsin C.H., Chuang C.Y. (2017). Bacterial alterations in salivary microbiota and their association in oral cancer. Sci. Rep..

[102] Guerrero-Preston R., White J.R., Godoy-Vitorino F., Rodríguez-Hilario A., Navarro K., González H., Michailidi C., Jedlicka A., Canapp S., Bondy J. (2017). High-resolution microbiome profiling uncovers *Fusobacterium nucleatum*, *Lactobacillus gasseri/johnsonii*, and *Lactobacillus vaginalis* associated to oral and oropharyngeal cancer in saliva from HPV positive and HPV negative patients treated with surgery and chemo-radiation. Oncotarget.

[103] Vesty A., Gear K., Biswas K., Radcliff F.J., Taylor M.W., Douglas R.G. (2018). Microbial and inflammatory-based salivary biomarkers of head and neck squamous cell carcinoma. Clin. Exp. Dent. Res..

[104] Hsiao J.R., Chang C.C., Lee W.T., Huang C.C., Ou C.Y., Tsai S., Chen K.C., Huang J.S., Wong T.Y., Lai Y.H. (2018). The interplay between oral microbiome, lifestyle factors and genetic polymorphisms in the risk of oral squamous cell carcinoma. Carcinogenesis.

[105] Hashimoto K., Shimizu D., Hirabayashi S., Ueda S., Miyabe S., Oh-Iwa I., Nagao T., Shimozato K., Nomoto S. (2019). Changes in oral microbial profiles associated with oral squamous cell carcinoma vs leukoplakia. J. Investig. Clin. Dent..

[106] Takahashi Y., Park J., Hosomi K., Yamada T., Kobayashi A., Yamaguchi Y., Iketani S., Kunisawa J., Mizuguchi K., Maeda N. (2019). Analysis of oral microbiota in Japanese oral cancer patients using 16S rRNA sequencing. J. Oral Biosci..

[107] Li Y., Tan X., Zhao X., Xu Z., Dai W., Duan W., Huang S., Zhang E., Liu J., Zhang S. (2020). Composition and function of oral microbiota between gingival squamous cell carcinoma and periodontitis. Oral Oncol..

[108] Gopinath D., Kunnath Menon R., Chun Wie C., Banerjee M., Panda S., Mandal D., Behera P.K., Roychoudhury S., Kheur S., George Botelho M. (2020). Salivary bacterial shifts in oral leukoplakia resemble the dysbiotic oral cancer bacteriome. J. Oral Microbiol..

[109] Zuo H.J., Fu M.R., Zhao H.L., Du X.W., Hu Z.Y., Zhao X.Y., Ji X.Q., Feng X.Q., Zhumajiang W., Zhou T.H. (2020). Study on the Salivary Microbial Alteration of Men with Head and Neck Cancer and Its Relationship with Symptoms in Southwest China. Front. Cell. Infect. Microbiol..

[110] Rai A.K., Panda M., Das A.K., Rahman T., Das R., Das K., Sarma A., Kataki A.C., Chattopadhyay I. (2021). Dysbiosis of salivary microbiome and cytokines influence oral squamous cell carcinoma through inflammation. Arch. Microbiol..

[111] Mohamed N., Litlekalsøy J., Ahmed I.A., Martinsen E.M.H., Furriol J., Javier-Lopez R., Elsheikh M., Gaafar N.M., Morgado L., Mundra S. (2021). Analysis of Salivary Mycobiome in a Cohort of Oral Squamous Cell Carcinoma Patients From Sudan Identifies Higher Salivary Carriage of Malassezia as an Independent and Favorable Predictor of Overall Survival. Front. Cell. Infect. Microbiol..

[112] Zhou X., Hao Y., Peng X., Li B., Han Q., Ren B., Li M., Li L., Li Y., Cheng G. (2021). The Clinical Potential of Oral Microbiota as a Screening Tool for Oral Squamous Cell Carcinomas. Front. Cell. Infect. Microbiol..

[113] Torralba M.G., Aleti G., Li W., Moncera K.J., Lin Y.H., Yu Y., Masternak M.M., Golusinski W., Golusinski P., Lamperska K. (2021). Oral Microbial Species and Virulence Factors Associated with Oral Squamous Cell Carcinoma. Microb. Ecol..

[114] Granato D.C., Neves L.X., Trino L.D., Carnielli C.M., Lopes A.F.B., Yokoo S., Pauletti B.A., Domingues R.R., Sá J.O., Persinoti G. (2021). Meta-omics analysis indicates the saliva microbiome and its proteins associated with the prognosis of oral cancer patients. Biochim. Biophys. Acta Proteins Proteom..

[115] Mauceri R., Coppini M., Vacca D., Bertolazzi G., Cancila V., Tripodo C., Campisi G. (2023). No Clear Clustering Dysbiosis from Salivary Microbiota Analysis by Long Sequencing Reads in Patients Affected by Oral Squamous Cell Carcinoma: A Single Center Study. Cancers.

[116] Mäkinen A.I., Pappalardo V.Y., Buijs M.J., Brandt B.W., Mäkitie A.A., Meurman J.H., Zaura E. (2023). Salivary microbiome profiles of oral cancer patients analyzed before and after treatment. Microbiome.

[117] Muto M., Hitomi Y., Ohtsu A., Shimada H., Kashiwase Y., Sasaki H., Yoshida S., Esumi H. (2000). Acetaldehyde production by non-pathogenic Neisseria in human oral microflora: Implications for carcinogenesis in upper aerodigestive tract. Int. J. Cancer.

[118] Chen Q., Shao Z., Liu K., Zhou X., Wang L., Jiang E., Luo T., Shang Z. (2021). Salivary Porphyromonas gingivalis predicts outcome in oral squamous cell carcinomas: A cohort study. BMC Oral Health.

[119] Michikawa C., Gopalakrishnan V., Harrandah A.M., Karpinets T.V., Garg R.R., Chu R.A., Park Y.P., Chukkapallia S.S., Yadlapalli N., Erikson-Carter K.C. (2022). Fusobacterium is enriched in oral cancer and promotes induction of programmed death-ligand 1 (PD-L1). Neoplasia.

[120] Harrandah A.M., Chukkapalli S.S., Bhattacharyya I., Progulske-Fox A., Chan E.K.L. (2020). Fusobacteria modulate oral carcinogenesis and promote cancer progression. J. Oral Microbiol..

[121] Perera M., Al-Hebshi N.N., Speicher D.J., Perera I., Johnson N.W. (2016). Emerging role of bacteria in oral carcinogenesis: A review with special reference to perio-pathogenic bacteria. J. Oral Microbiol..

[122] Karpiński T.M. (2019). Role of Oral Microbiota in Cancer Development. Microorganisms.

[123] Wei J., Xie G., Zhou Z., Shi P., Qiu Y., Zheng X., Chen T., Su M., Zhao A., Jia W. (2011). Salivary Metabolite Signatures of Oral Cancer and Leukoplakia. Int. J. Cancer.

[124] Vimal J., George N.A., Kumar R.R., Kattoor J., Kannan S. (2023). Identification of salivary metabolic biomarker signatures for oral tongue squamous cell carcinoma. Arch. Oral Biol..

[125] Tantray S., Sharma S., Prabhat K., Nasrullah N., Gupta M. (2022). Salivary metabolite signatures of oral cancer and leukoplakia through gas chromatography-mass spectrometry. J. Oral Maxillofac. Pathol..

[126] Ishikawa S., Sugimoto M., Konta T., Kitabatake K., Ueda S., Edamatsu K., Okuyama N., Yusa K., Iino M. (2022). Salivary Metabolomics for Prognosis of Oral Squamous Cell Carcinoma. Front. Oncol..

[127] de Sá Alves M., de Sá Rodrigues N., Bandeira C.M., Chagas J.F.S., Pascoal M.B.N., Nepomuceno G.L.J.T., da Silva Martinho H., Alves M.G.O., Mendes M.A., Dias M. (2021). Identification of Possible Salivary Metabolic Biomarkers and Altered Metabolic Pathways in South American Patients Diagnosed with Oral Squamous Cell Carcinoma. Metabolites.

[128] Yatsuoka W., Ueno T., Miyano K., Enomoto A., Ota S., Sugimoto M., Uezono Y. (2021). Time-Course of Salivary Metabolomic Profiles during Radiation Therapy for Head and Neck Cancer. J. Clin. Med..

[129] Supawat B., Aye K.T., Ritpanja J., Nueangwong W., Kothan S., Pan J., Tungjai M. (2021). Differences in Spectroscopic Properties of Saliva Taken From Normal Subjects and Oral Cancer Patients: Comparison Studies. J. Fluoresc..

[130] Song X., Yang X., Narayanan R., Shankar V., Ethiraj S., Wang X., Duan N., Ni Y.-H., Hu Q., Zare R.N. (2020). Oral Squamous Cell Carcinoma Diagnosed from Saliva Metabolic Profiling. Proc. Natl. Acad. Sci. USA.

[131] Ishikawa S., Wong D.T.W., Sugimoto M., Gleber-Netto F.O., Li F., Tu M., Zhang Y., Akin D., Iino M. (2019). Identification of Salivary Metabolites for Oral Squamous Cell Carcinoma and Oral Epithelial Dysplasia Screening from Persistent Suspicious Oral Mucosal Lesions. Clin. Oral Investig..

[132] Shigeyama H., Wang T., Ichinose M., Ansai T., Lee S.-W. (2019). Identification of Volatile Metabolites in Human Saliva from Patients with Oral Squamous Cell Carcinoma via Zeolite-Based Thin-Film Microextraction Coupled with GC-MS. J. Chromatogr. B Analyt. Technol. Biomed. Life Sci..

[133] Sridharan G., Ramani P., Patankar S., Vijayaraghavan R. (2019). Evaluation of Salivary Metabolomics in Oral Leukoplakia and Oral Squamous Cell Carcinoma. J. Oral Pathol. Med. Off. Publ. Int. Assoc. Oral Pathol. Am. Acad. Oral Pathol..

[134] Lohavanichbutr P., Zhang Y., Wang P., Gu H., Nagana Gowda G.A., Djukovic D., Buas M.F., Raftery D., Chen C. (2018). Salivary Metabolite Profiling Distinguishes Patients with Oral Cavity Squamous Cell Carcinoma from Normal Controls. PLoS ONE.

[135] Mikkonen J.J.W., Singh S.P., Akhi R., Salo T., Lappalainen R., González-Arriagada W.A., Ajudarte Lopes M., Kullaa A.M., Myllymaa S. (2018). Potential Role of Nuclear Magnetic Resonance Spectroscopy to Identify Salivary Metabolite Alterations in Patients with Head and Neck Cancer. Oncol. Lett..

[136] Taware R., Taunk K., Pereira J.A.M., Shirolkar A., Soneji D., Câmara J.S., Nagarajaram H.A., Rapole S. (2018). Volatilomic Insight of Head and Neck Cancer via the Effects Observed on Saliva Metabolites. Sci. Rep..

[137] Ohshima M., Sugahara K., Kasahara K., Katakura A. (2017). Metabolomic Analysis of the Saliva of Japanese Patients with Oral Squamous Cell Carcinoma. Oncol. Rep..

[138] Ishikawa S., Sugimoto M., Kitabatake K., Sugano A., Nakamura M., Kaneko M., Ota S., Hiwatari K., Enomoto A., Soga T. (2016). Identification of Salivary Metabolomic Biomarkers for Oral Cancer Screening. Sci. Rep..

[139] Wang Q., Gao P., Cheng F., Wang X., Duan Y. (2014). Measurement of Salivary Metabolite Biomarkers for Early Monitoring of Oral Cancer with Ultra Performance Liquid Chromatography-Mass Spectrometry. Talanta.

[140] Wang Q., Gao P., Wang X., Duan Y. (2014). Investigation and Identification of Potential Biomarkers in Human Saliva for the Early Diagnosis of Oral Squamous Cell Carcinoma. Clin. Chim. Acta Int. J. Clin. Chem..

[141] Wang Q., Gao P., Wang X., Duan Y. (2014). The Early Diagnosis and Monitoring of Squamous Cell Carcinoma via Saliva Metabolomics. Sci. Rep..

[142] Sugimoto M., Wong D.T., Hirayama A., Soga T., Tomita M. (2010). Capillary Electrophoresis Mass Spectrometry-Based Saliva Metabolomics Identified Oral, Breast and Pancreatic Cancer-Specific Profiles. Metab. Off. J. Metab. Soc..

[143] Rai B., Kharb S., Jain R., Anand S.C. (2007). Salivary Vitamins E and C in Oral Cancer. Redox. Rep..

[144] Zabokova Bilbilova E., Sotirovska Ivkovska A., Ambarkova V. (2012). Correlation between salivary urea level and dental caries. Prilozi.

[145] Ma Z., Vosseller K. (2014). Cancer metabolism and elevated O-GlcNAc in oncogenic signaling. J. Biol. Chem..

[146] Gatenby R.A., Gillies R.J. (2004). Why do cancers have high aerobic glycolysis?. Nat. Rev. Cancer.

[147] Garritano S., Inga A., Gemignani F., Landi S. (2013). More targets, more pathways and more clues for mutant p53. Oncogenesis.

[148] Chuchueva N., Carta F., Nguyen H.N., Luevano J., Lewis I.A., Rios-Castillo I., Fanos V., King E., Swistushkin V., Reshetov I. (2023). Metabolomics of head and neck cancer in biofluids: An integrative systematic review. Metabolomics.

[149] Ogretmen B. (2018). Sphingolipid metabolism in cancer signalling and therapy. Nat. Rev. Cancer.

[150] Dickinson A., Saraswat M., Joenväärä S., Agarwal R., Jyllikoski D., Wilkman T., Mäkitie A., Silén S. (2020). Mass spectrometry-based lipidomics of oral squamous cell carcinoma tissue reveals aberrant cholesterol and glycerophospholipid metabolism—A Pilot study. Transl. Oncol..

[151] Zhang G., Chen R., Rudney J.D. (2011). Streptococcus cristatus modulates the Fusobacterium nucleatum-induced epithelial interleukin-8 response through the nuclear factor-kappa B pathway. J. Periodontal. Res..

[152] Nobbs A.H., Jenkinson H.F., Jakubovics N.S. (2011). Stick to your gums: Mechanisms of oral microbial adherence. J. Dent. Res..

[153] Gur C., Ibrahim Y., Isaacson B., Yamin R., Abed J., Gamliel M., Enk J., Bar-On Y., Stanietsky-Kaynan N., Coppenhagen-Glazer S. (2015). Binding of the Fap2 protein of Fusobacterium nucleatum to human inhibitory receptor TIGIT protects tumors from immune cell attack. Immunity.

[154] Binder Gallimidi A., Fischman S., Revach B., Bulvik R., Maliutina A., Rubinstein A.M., Nussbaum G., Elkin M. (2015). Periodontal pathogens Porphyromonas gingivalis and Fusobacterium nucleatum promote tumor progression in an oral-specific chemical carcinogenesis model. Oncotarget.

[155] Schwabe R.F., Jobin C. (2013). The microbiome and cancer. Nat. Rev. Cancer.

[156] Pang X., Tang Y.J., Ren X.H., Chen Q.M., Tang Y.L., Liang X.H. (2018). Microbiota, Epithelium, Inflammation, and TGF-β Signaling: An Intricate Interaction in Oncogenesis. Front. Microbiol..

[157] Al-Hebshi N.N., Nasher A.T., Maryoud M.Y., Homeida H.E., Chen T., Idris A.M., Johnson N.W. (2017). Inflammatory bacteriome featuring *Fusobacterium nucleatum* and *Pseudomonas aeruginosa* identified in association with oral squamous cell carcinoma. Sci. Rep..

[158] Kouznetsova V.L., Li J., Room E., Tsigelny I.F. (2021). Finding distinctions between oral cancer and periodontitis using saliva metabolites and machine learning. Oral Dis..

[159] Gawron K., Wojtowicz W., Łazarz-Bartyzel K., Łamasz A., Qasem B., Mydel P., Chomyszyn-Gajewska M., Potempa J., Mlynarz P. (2019). Metabolomic status of the oral cavity in chronic periodontitis. In Vivo.

[160] Liu B., Faller L.L., Klitgord N., Mazumdar V., Ghodsi M., Sommer D.D., Gibbons T.R., Treangen T.J., Chang Y.C., Li S. (2012). Deep sequencing of the oral microbiome reveals signatures of periodontal disease. PLoS ONE.

[161] Komarova T.V., Petrunia I.V., Shindyapina A.V., Silachev D.N., Sheshukova E.V., Kiryanov G.I., Dorokhov Y.L. (2014). Endogenous methanol regulates mammalian gene activity. PLoS ONE.

[162] Maitre Y., Mahalli R., Micheneau P., Delpierre A., Guerin M., Amador G., Denis F. (2021). Pre and Probiotics Involved in the Modulation of Oral Bacterial Species: New Therapeutic Leads in Mental Disorders?. Microorganisms.

